# PD-L1 as a Prognostic Factor in Early-Stage Colon Carcinoma within the Immunohistochemical Molecular Subtype Classification

**DOI:** 10.3390/cancers13081943

**Published:** 2021-04-17

**Authors:** Pablo Azcue, Ignacio Encío, David Guerrero Setas, Javier Suarez Alecha, Arkaitz Galbete, María Mercado, Ruth Vera, Maria Luisa Gomez-Dorronsoro

**Affiliations:** 1 Department of Health Science, Public University of Navarra (UPNA), 31008 Pamplona, Spain; ignacio.encio@unavarra.es; 2 Institute for Health Research Navarra (IdISNA), 31008 Pamplona, Spain; arkaitz.galbete@navarra.es (A.G.); ruth.vera.garcia@navarra.es (R.V.); 3 Department of Molecular Pathology, Hospital Complex of Navarra (CHN), 31008 Pamplona, Spain; dguerres@navarra.es (D.G.S.); mr.mercado.gutierrez@navarra.es (M.M.); 4 Campus Arrosadia, Public University of Navarra (UPNA), 31006 Pamplona, Spain; 5 Molecular Pathology of Cancer Group–Navarrabiomed, 31008 Pamplona, Spain; 6 Department of Medical Oncology, Hospital Complex of Navarra (CHN), 31008 Pamplona, Spain; 7 Department of Surgery, Hospital Complex of Navarra (CHN), 31008 Pamplona, Spain; fj.suarez.alecha@navarra.es; 8 Navarrabiomed-Hospital Complex of Navarra (CHN), Redissec, 31008 Pamplona, Spain

**Keywords:** PD-L1, CMS, colon cancer

## Abstract

**Simple Summary:**

Colorectal cancer (CRC) is a very heterogeneous disease. Efforts to characterize and search for biomarkers for these patients are currently ongoing in the hope of establishing a more targeted therapeutic approach. The role of PD-1 ligand (PD-L1) expression as a biomarker has not yet been fully elucidated. The Consensus Molecular Subtype classification has been delineated, but although already acknowledged in the most recent international guidelines, it has yet to be implemented in clinical practice. We investigate PD-L1 expression as a biomarker of prognosis in the early-stage setting and integrate it with the Consensus Molecular Subtype (CMS), in an effort to differentiate those patients with a worse prognosis who could potentially benefit from an early, more aggressive treatment. Our results suggest PD-L1 as an independent prognostic factor in early stage setting when assessed by immunohistochemistry. Additionally, PD-L1 expression appears to be a viable biomarker to differentiate patients in the CMS (CMS2/CMS3) who lack a clear prognosis.

**Abstract:**

Background. There is a patent need to better characterize early-stage colorectal cancer (CRC) patients. PD-1 ligand (PD-L1) expression has been proposed as a prognostic factor but yields mixed results in different settings. The Consensus Molecular Subtype (CMS) classification has yet to be integrated into clinical practice. We sought to evaluate the prognostic value of PD-L1 expression overall and within CMS in early-stage colon cancer patients, in the hope of assisting treatment choice in this setting. Methods. Tissue-microarrays were constructed from tumor samples of 162 stage II/III CRC patients. They underwent automatic immunohistochemical staining for PD-L1 and the proposed CMS panel. Primary endpoints were overall survival (OS) and disease-free survival (DFS). Results. PD-L1 expression was significantly and independently associated with better prognosis (HR = 0.46 (0.26–0.82), *p* = 0.009) and was mostly seen in immune cells of the tumor-related stroma. CMS4 five-folds the risk of mortalitycompared with CMS1 (HR = 5.58 (1.36, 22.0), *p* = 0.034). In the subgroup CMS2/CMS3 analysis, PD-L1 expression significantly differentiated individuals with better OS (*p* = 0.004) and DFS (*p* < 0.001). Conclusions. Our study suggests that PD-L1 expression is an independent prognostic factor in patients with stage II/III colon cancer. Additionally, it successfully differentiates patients with better prognosis in the CMS2/CMS3 group and may prove significant for the clinical relevance of the CMS classification.

## 1. Introduction

Colorectal cancer (CRC) is currently the third most commonly diagnosed cancer and the second leading cause of cancer death worldwide [1], due in part to it being a highly heterogeneous disease. A classification of CRC patients is needed to provide the basis for better treatment decisions and targeted therapies, particularly in early-stage settings [2]. Through transcriptomics the Consensus Molecular Subtype (CMS) classification has been delineated and proposed as a prognostic tool with predictive capabilities and therapeutic implications [3]. However, these types of techniques require specialized resources that are not within the reach of most hospitals.

In a first effort to address this issue, a subrogated panel of four proteins (CDX2, FRMD6, HTR2B and ZEB1) has been validated through immunohistochemistry (IHC) [4]. This panel allows a classification of CRC patients that is reproducible and more easily accessible to hospitals and laboratories. The proposed panel classifies patients into CMS4 and CMS2/CMS3 subtypes, with CMS1 being defined by the mismatch repair (MMR) proteins panel: CDX2 is a homeobox transcription factor expressed in early intestinal development, where it regulates proliferation, differentiation, cell adhesion and migration of intestinal epithelial cells [5]. Pilati et al. reported that lack of CDX2 expression in the CMS classification is useful for identifying poor prognosis patients (CMS4/CDX2-negative), whereas CMS2 and CMS3 tumors rarely show total loss of CDX2 [6]. HTR2B is a G-protein coupled receptor subtype of the serotonin family that is overexpressed in various solid tumors [7,8] and has a higher level of expression in mesenchymal-like tumors [4]. FRMD6 is an Ezrin/Radixin/Moesin family protein that is part of the Hippo signaling pathway kinase cascade [9]. Its loss of expression contributes to the epithelial-to-mesenchymal transition (EMT) while its overexpression antagonizes the yes-associated protein 1 (YAP) activity [10]. ZEB1 is a transcription factor regulated by a variety of signaling pathways including WNT [11]. It promotes invasion and metastasis by inducing EMT and is frequently observed in mesenchymal-like carcinoma cells that confer resistance to cancer therapy [12].

MMR protein expression (MLH1, MSH2, MSH6, PMS2) is studied to determine microsatellite instability (MSI) or deficient MMR (dMMR), which accounts for 15–20% of CRCs [13,14,15]. In the current ESMO and NCCN colon cancer guidelines, dMMR status is acknowledged as being a valid prognostic biomarker of CRC in some settings, although other major and minor prognostic tools, especially TNM staging, must be used when deciding whether to offer adjuvant therapy [16,17]. Recently, anti-PD1 treatment has proven to be beneficial in MSI high patients [18].

The aforementioned four-biomarker IHC panel and the MMR panel can identify the CMS4 and CMS1 subtypes, which have the worst and best prognosis, respectively. CMS2 and CMS3 account for more than 50% of the population and are indistinguishable from each other by these panels. This CMS2/CMS3 group includes patients with very different molecular characteristics [19] and survival [20,21,22,23]. Therefore, there is a need for new biomarkers that can provide clear expectations about prognosis, particularly for this group of patients.

Programmed cell death protein 1 (PD-1, also known as PDCD1 and CD279) is an inhibitory receptor that is expressed by T cells during activation. It regulates T cell effector functions during various physiological responses, including acute and chronic infection, cancer and autoimmunity, and in immune homeostasis [24]. Some cancer cells can develop PD-1 ligand (PD-L1) expression, which potentially shields it from immune attack by inhibiting T cell effector functions [25]. Its expression has been associated with the serrated pathway of colorectal carcinogenesis, with the presence of BRAF mutation, dMMR and poor differentiation [26]. The potential activation of the WNT/β-catenin pathway by this receptor has also been linked to progression [27]. Additionally, a recent study has reported the regulation of PD-L1 by the Zinc finger E-box binding homeobox 1 (ZEB1), an EMT inhibitor [28].

PD-L1 expression has been proposed as being a biomarker of prognosis in early CRC, but has yet to be fully devised, probably due to the lack of standardization in IHC assessment and homogenization for the studied population [29].

PD-L1 was first studied as a predictive tool in CRC, although early published studies yielded some apparently contradictory results [26,30,31]. More recently, it has proved to be of predictive value for anti-PD-1 therapy for overall survival (OS), and especially for overall response rate (ORR) and progression-free survival (PFS) [18,32]. This has been achieved mainly by post hoc analysis, which proposes, among other things, a different cut-off value for PD-L1 expression and a different methodology for the pathological assessment (>1%, >5%, >50%) [25,29,33,34].

We hypothesize that PD-L1 expression, when assessed by IHC using a standardized methodology, is a strong candidate for assessment as a potential biomarker. Furthermore, when added to the panel for CMS classification, it could prove helpful for patients whose immune response is not as clear as in those of the CMS2/CMS3 subtypes. Therefore, the objective is to first corroborate the possibility of classifying a large cohort of early-stage CRC patients into CMS subtypes through the proposed IHC panel, and then to investigate the prognostic role of PD-L1 expression in addition to the previous panel, specifically for patients in the CMS2/CMS3 subgroup. We expect to obtain clearer expectations about the CMS2/CMS3 subgroup that might inform physicians’ choice of treatment for early-stage patients in routine clinical practice.

## 2. Materials and Methods

This study was performed in accordance with the World Medical Association Declaration of Helsinki. The study was approved by the Regional Clinical Research Ethics Committee (CEIC) Pyto2017/51 Cod. MOL_CRC, 15 May 2018. Patient consent was waived due to the use of stored tumor samples for research purposes in compliance with the current Spanish and European Union legislation (resolution 1387/2017 (08/11) and resolution 193/2018 (06/03) of the Navarra Health Service—Osasunbidea).

### 2.1. Patients

The cohort of this retrospective study consists of 162 patients diagnosed with stage II/III CRC consecutively surgically resected with curative intention in the Hospital Complex of Navarra between 2009 and 2013. All patients were diagnosed by the Department of Pathology, following the standardized treatment protocol established by the Colorectal Committee. Participants were then followed until death or last medical consultation, with a cut-off date of 1 October 2018, when the clinical data were retrieved, anonymized and analyzed.

The clinical follow-up protocol included a medical visit and carcinoembryonic antigen monitoring every three months for two years and then every six months for three more years. Computed tomography (CT) was performed annually, at the same time in years one and five as a colonoscopy. A CT of the abdomen and chest x-ray or CT was performed preoperatively to rule out distant metastases in all patients. The data retrieved subsequently included age, gender, localization (the right and left colons were, respectively, defined as proximal and distal to the splenic angle [35]), differentiation grade (defined as exhibiting less than 50% or at least 50% of glandular formations), lymph node ratio, histological type, and lymphatic, blood vessel and perineural invasion. Tumors were classified according to the TNM Classification of Malignant Tumors (TNM), 7th edition [36].

Only patients with stage II/III CRC and a confirmed pathological diagnosis of adenocarcinoma were included in the study. Patients who had insufficient tumor material, were lost to follow-up for at least three years or had died, had fewer than two IHC-stained blocks for evaluation, or whose information about their baseline characteristics was missing were then excluded. To further homogenize the study population, we decided to include only cases with colon carcinoma (CC).

One hundred and forty-four patients from the original cohort were included in the statistical analyses.

### 2.2. Pathological Study

Hematoxylin-eosin sections representative of the invasive carcinoma from the Formalin-fixed paraffin-embedded (FFPE) tissue specimens were selected for each patient. Four spots/areas were annotated per case in the infiltrating tumor, two from the tumor periphery or invasion front, and two from the central tumor area to minimize the heterogeneity within the tumor. Areas of abscess or necrosis were avoided.

Corresponding donor tissue cores were then transferred to the tissue microarray (TMA) recipient blocks using a manual Tissue Arrayer MTA-1 (Beecher Instruments Silver Spring, MD, USA). Four representative 1-mm-diameter cores were obtained in sequence for each tumor after confirmation of each annotation in selected areas. Each TMA block consisted of two sections containing 10 × 5 cores, and four tissue cores from benign colon and ovary tissue selected as controls and for orientation (Figure S1). Each TMA block was divided into two halves: the first half contained a pair of consecutive TMA samples for each patient; the second half also contained the second pair of consecutive TMA samples but in a different order, with each half containing a control. This method was adopted to reduce possible evaluator bias when analyzing consecutive samples from the same patient.

The constructed TMAs blocks were then sectioned in 4μm slides, stained, scanned and finally scored as described below.

### 2.3. Immunohistochemical Analysis

The TMAs sections underwent immunohistochemical staining against CDX2, FRMD6, HTR2B, and ZEB1 for the CMS classification. This IHC-based screening panel was used as a surrogate for gene expression profiling [37].

The antibodies used were anti-FRMD6 (Clone HPA001297; 1:50; Sigma), anti-HTR2B (Clone HPA012867; 1:50; Sigma), anti-ZEB1 (Clone HPA027524; 1:50; Sigma), using Roche’s BenchMark Ventana automatic immunostainer. The anti-CDX2 (Clone PA0535; RTU; Novocastra), anti-cytokeratin (Clone PA0909; RTU; Leica), and the MMR proteins MLH1 (Clone PA0610; RTU; Biocare), MSH2 (Clone FE-11; 1:100; Calbiochem), MSH6 (Clone PM265AA; RTU; Biocare) and PMS2 (Clone PM344AA; RTU; Biocare), were used to determine dMMR status using Leica Biosystems’ Bond automatic immunostainer, and the BRAF V600E mutation was determined through anti-BRAF (Clone VE1, 1:1, Roche) by IHC.

Finally, to determine PD-L1 expression, TMAs underwent staining using the antibody anti-PD-L1 (SP142; RTU; Roche) following the specifically approved protocol. Staining was performed in each section, after antigenic recovery and endogen peroxidase blockage, by sequentially incubating the specific primary and secondary antibodies, and revealed with the Optiview Universal DAB Detection Kit using an automatic BenchMark XT (VENTANA/Roche).

Each stained TMA array was then scanned and digitalized using the VENTANA iScan HT Slide scanner. Images were processed using the integrated Virtuoso image and workflow management software (VENTANA/Roche).

### 2.4. IHC Scoring and Evaluation

Once digitized, each individual sample from the TMA slide was scored by two independent evaluators (a trained senior scientist and an expert pathologist), both of whom were blinded to the patients’ clinical data. In the event of discordant results, a wash-out period of three weeks was imposed, after which the evaluators scored the samples again and, with the aid of reference images of each antibody, arrived at a consensus score.

The CMS assessment was assessed according to published methodology [37]. The scores obtained were then uploaded to the online IHC classifier (https://crcclassifier.shinyapps.io/appTesting, accessed on: 2 September 2020) and the CMS2/CMS3 and CMS4 subtypes were established.

The status of MMR was determined as proficient (pMMR) or deficient (dMMR). A case was considered to be pMMR when any focus of the tumor exhibited positive nuclear staining for all MMR proteins (MLH1, PMS2, MSH2 and MSH6). If the tumor showed a total loss of staining for any of these proteins in all tumor cells, it was considered to be dMMR. The latter were first used to define patients as belonging to the CMS1 subtype. Lymphocytes were used as an internal control for evidence of positive staining. BRAF V600E was classified dichotomously as mutated (pathological) or wild type, also with a known mutated colon adenocarcinoma control for positive staining.

The expression of PD-L1 was measured when at least 50 viable tumor cells were present. A sample of the amygdala was used as a control in each TMA. For the IHC assessment, the SP142 antibody guidelines state that the determination of PD-L1 status evaluation is based on the percentage area of positive immune cells within the total area of inflammation and tumor-related stroma (%) of any intensity and the percentage area of PD-L1 expressing tumor cells within the total tumor area (%) of any intensity [29,38].

**Figure 1:**
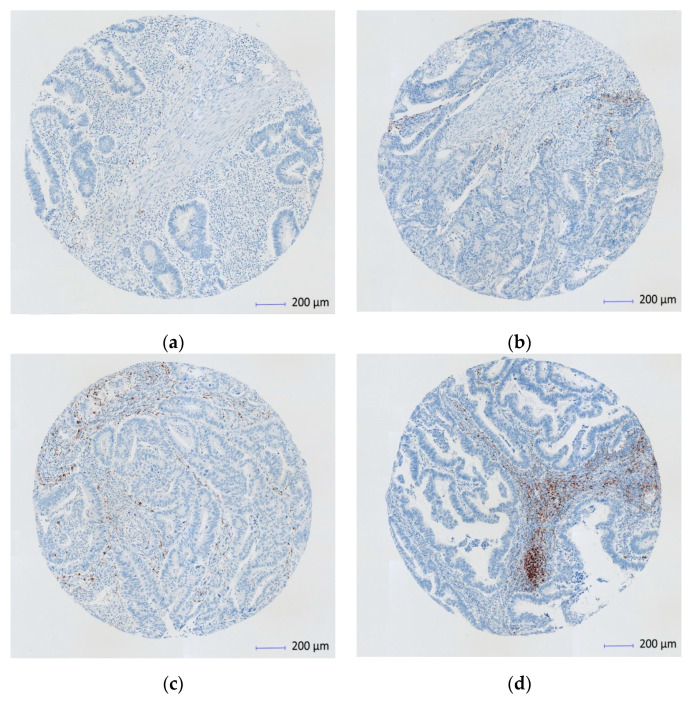
Immunohistochemistry (IHC) scan of PD-1 ligand (PD-L1) expression on immune cells at the tumor-stroma interface (Scanned images core X4). (**a**) PD-L1 < 1% (score 0); (**b**) PD-L1 of > 1–5% (score 1); (**c**) PD-L1 > 5%–< 50% (score 2); (**d**) PD-L1 > 50% (score 3).

In our study, the presence of discernible PD-L1 staining of any intensity was discernible in immune cells (lymphocytes, macrophages and dendritic cells) in the tumor-related stroma (Figure 1). The assessment was performed using a four-level score based on the percentage stained, as follows: 0 when <1%; 1 between 1% and <5%; 2 between 5% and <50%; and 3 when >50%. To maximize sensitivity and specificity, a score of 0 was considered as negative, 1 or more as low expression (PD-L1–L) and 2 or more as high expression (PD-L1–H).

### 2.5. Statistical Analysis

The primary endpoint OS was defined as time from surgery to death due to any cause and disease-free survival (DFS) was defined as time from surgery to relapse or death due to any cause. A predetermined subgroup analysis of OS and DFS for the expression of PD-L1 in the CMS2/CMS3 population was performed.

The statistical analysis was performed by using the SPSS 24.0 software (IBM, New York, USA). Associations between variables among groups were determined using the *t*-test, Mann–Whitney U test or ANOVA for quantitative variables and using Fisher’s exact test and the χ² test for categorical ones. Univariate and multivariate Cox proportional hazard regression models were used for OS and DFS analysis. The multivariate model was adjusted for the factors that proved significant with survival in the univariate analysis, which included baseline clinical variables such as age and sex. The survival curves were calculated using the Kaplan–Maier method and the log-rank test. Statistical significance was set at two tailed *p*-value of <0.05.

## 3. Results

To assess the prognostic value of PD-L1 expression in addition to the CMS classification in early-stage CC patients, 144 patients were analyzed. Only patients with colon adenocarcinomas were included in the analysis. The majority of patients were men (68.1%), with stage II (55.6%), well or moderately differentiated (81.9%) and right-sided (54.9%) tumors. Patient baseline characteristics and main clinical parameters are summarized in Table 1.

In the IHC analysis CMS2/CMS3 represents the largest subgroup (81.3%), whereas the CMS1/dMMR subgroup represented 12.5% and CMS4 made up 6.3% of the cohort. The expression of PD-L1–L (more than 1% of immune cells) was present in 55.5% of patients, and PD-L1–H (more than 5% of immune cells) was present in 20.1% of patients. Distributions of CMS subtypes, PD-L1 expression, BRAF expression and MMR protein deficiency, according to IHC analysis, are presented in Table 2.

Patients were followed for a median of 65.0 months (95% CI (62.2–67.7)), during which time 27 patients (18.8%) relapsed and 51 patients (35.4%) died. The number of outcome events (relapse and death, respectively) in the CMS subgroups were as follows: CMS1 subgroup 1 (5.6%) and 4 (22.2%), CMS2/3 subgroup 22 (18.8%) and 42 (35.9%), CMS4 subgroup 4 (44.4%) and 5 (55.6%).

### 3.1. Comparative Analysis

Mismatch repair deficiency (dMMR) was significantly more frequent in the right-sided tumors (*p* = 0.002) and was also significantly more frequently associated with PD-L1 expression (*p* < 0.001).

The CMS classification showed a significant difference in the expression of both PD-L1–L (*p* = 0.038) and PD-L1–H (*p* < 0.001). The differences were mainly due to the overexpression in CMS1 and under expression in CMS4. For PD-L1–L the expression was found in 77.8% of the CMS1 group, in 22.2% of the CMS4 group and in almost half (49.6%) of the CMS2/CMS3 group.

A statistically significant difference was found for TNM stage and CMS (*p* = 0.016). Stage II and III patients were more frequently classified into CMS1 and CMS4, respectively, but similar numbers of stage II and III patients were classified as CMS2/CMS3. Differences were also found with respect to localization and CMS; right-sided tumors were more often classified into CMS1, whereas there were no differences for CMS2/CMS3.

PD-L1 expression was more often expressed in stage II tumors (*p* = 0.014) and was found concomitantly with BRAF mutation (*p* = 0.002). A full comparative analysis is presented in Table 3.

### 3.2. Univariate Analysis

The univariate analysis showed that the risk of mortality increased with age by about 9% per year (HR = 1.09 95% CI (1.04, 1.14)), *p* < 0.001), and, as expected, a relapse event significantly increased the risk of mortality (HR = 7.93 (95% CI 3.05, 20.6), *p* < 0.001), as seen in Table 4. Finally, perineural invasion also showed a tendency towards poor prognosis (HR = 2.20 (95% CI 0.99, 4.90), *p* = 0.050). The expression of PD-L1 was related to a reduced risk of death, especially for PD-L1–L (HR = 0.40 (95% CI 0.20, 0.81), *p* = 0.010). No significant difference was found in mortality between stage II and stage III patients (HR = 1.51 (95% CI 0.76, 2.99), *p* = 0.242).

### 3.3. Multivariate Analysis

The expression of PD-L1–L was associated with good prognosis in the univariate analysis and was confirmed as being independently associated with better OS in the multivariate analysis (HR = 0.46 (95% CI 0.26–0.82), *p* = 0.009) and DFS (HR = 0.48 (95% CI 0.28–0.83), *p* = 0.012). A high expression of PD-L1–H also showed a tendency towards statistical significance for better OS (HR = 0.42 (95% CI 0.17–1.02), *p* = 0.054) and DFS (HR = 0.46 (95% CI 0.20–1.05), *p* = 0.064).

CMS4 patients had five times greater risk of mortality and six times the risk of DFS compared to the CMS1 group (HR = 5.58 (95% CI 1.36, 22.0), *p* = 0.034 and HR = 6.33 (95% CI 1.68, 23.8), *p* = 0.012, respectively). CMS2/CM3 exhibited an intermediate prognosis with no statistically significant difference.

Independent variables associated with worse prognosis of mortality were age (HR = 1.09 (95% CI 1.05–1.13), *p* < 0.001) and perineural invasion (HR = 2.25 (95% CI 1.19–4.26), *p* = 0.012). Similar results for age and perineural invasion were found for DFS, but no significant differences were noted between the sexes.

### 3.4. Survival

**Figure 2:**
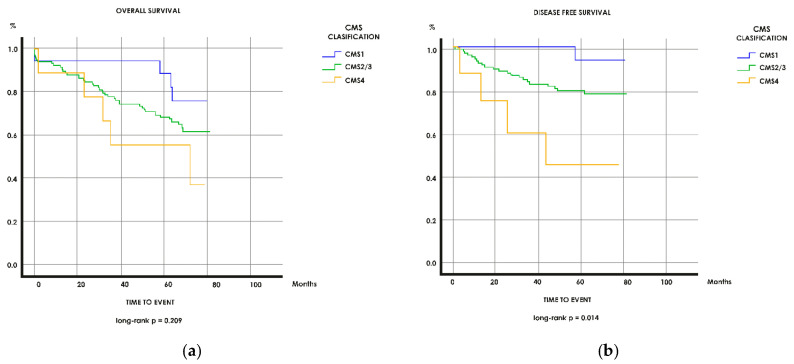

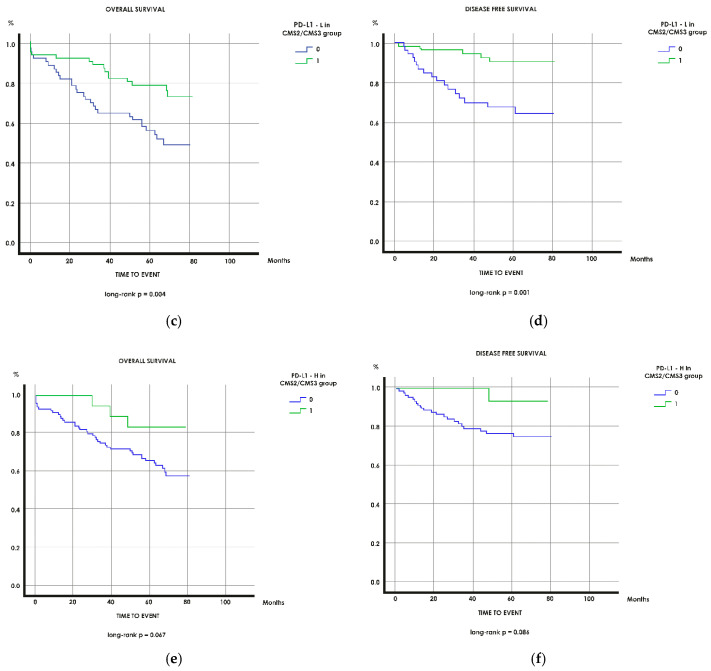
Survival analysis. Kaplan–Meier curves for (**a**) overall survival (OS) by CMS in the overall population; (**b**) disease-free survival (DFS) by CMS in the overall population; (**c**) OS for PD-L1–L in the CMS2/CMS3 group; (**d**) DFS for PD-L1–L in the CMS2/CMS3 group; (**e**) OS for PD-L1 – H in the CMS2/CMS3 group; (**f**) DFS for PD-L1–L in the CMS2/CMS3 group.

The Kaplan–Meier curves (Figure 2) for OS and DFS are consistent with previous results. With respect to OS and DFS, the CMS1 group displayed the longest survival, followed by the CMS2/CMS3 and finally the CMS4, which had the poorest outcome. In the subgroup analysis of CMS2/CMS3, PD-L1 expression significantly differentiated patients with good and poor prognosis for OS and time to relapse or death (*p* = 0.004 and *p* < 0.001, respectively).

## 4. Discussion

Our findings suggest that PD-L1 expression is an independent prognostic factor in patients with cancer in the CMS2/CMS3 group. Patients in this group with positive expression of PD-L1–L (≥1%) and of PD-L1–H (≥5%) in immune cells in tumor-related stroma had longer OS and DFS than patients with a lower or null level of expression. After adjustment for known clinical prognostic factors, the prognostic effect of PD-L1 remained significant in the multivariate analysis for both OS and DFS. The CMS1 group provided the best prognosis, whereas the CMS4 group exhibited the worst outcome.

Consistent with the findings of similar studies, patients with a diagnosis of rectal cancer were excluded from our analysis in an effort to homogenize the patient population, since rectal cancer differs from colon cancer with respect to the therapeutic approach, tumor biology and prognosis [29,39,40,41]. Furthermore, through the use of IHC, some studies have revealed elevated PD-L1 expression in rectal cancer after chemo-radiotherapy in the perioperative setting [42,43].

PD-L1 expression depends on various factors and their possible interactions, for example the type of tumor, pathological assessment, tumor stage, and technical issues related to IHC (e.g., the type of clone, scoring method, cut-off values for positivity, etc.). CRC is considered to be a cold tumor with a low PD-L1 expression compared with other solid tumors such as lung cancer, renal cell carcinoma and urothelial carcinoma. PD-L1 expression in CRC is not frequently observed in tumor cells [29,38,44,45,46], although this may not be the case for all clones. Accordingly, the PD-L1 expression in our study with the SP142 clone mostly occurred in the immune cells of the tumor-related stroma, and not in any tumor cells (Figure S2a,b). In a few cases, the expression was initially thought to occur in the tumor epithelium, but, on closer assessment, it was found to be due to infiltration of intratumoral lymphocytes [38]. In these few cases of intertumoral expression, they all co-existed with positivity at the tumor–stroma interface.

It has been suggested that overexpression of PD-L1 in CRC is fundamentally related to an extrinsic/adaptive mechanism that drives PD-L1 expression in immune cells, highlighting the role of the tumor microenvironment, rather than being associated with an intrinsic gene alteration [44,47,48,49]. One example is the MSI in CRC, where an “extrinsic” immune cell-mediated PD-L1 upregulation mechanism has been hypothesized to be exerted by the induction of an active immune microenvironment by this instability on two fronts: an immune-stimulatory effect by increased cytotoxic effector T lymphocytes on one side, and immune inhibitory effect that includes PD-1/PD-L1 checkpoint on the other [38,44,50]. Likewise, our results showed that dMMR tumors were significantly associated with PD-L1 expression in immune cells. Furthermore, the level of expression of PD-L1 was also significantly related to dMMR tumors since more cases were assessed as being PD-L1–H than PD-L1–L (34.5% vs. 17.5%). The exosomes are another example supporting the “extrinsic/adaptive” mechanism. As recently reported by Tang et al., exosomes may play a role in immunosuppression and avoiding an anti-tumor immune response [51]. Overall, it has been suggested that there is a lack of evidence supporting “intrinsic” mechanisms in CRC, unlike other solid tumors [38].

We used the SP142 clone because it has proved useful in other tumor types with clinical implications and with a particular sensitivity of expression in immune cells (e.g., breast, urothelial and non-small cell lung cancer [52,53,54,55]). Special attention is required with the scoring method and the cut-off values defining positivity when comparing results, since there is no established consensus. Contradictory results can be found in other studies using different cut-off levels to determine the scoring method and PD-L1 positivity [49,56,57]. However, similar studies concur in setting the low level of expression of PD-L1 (PD-L1–L) as ≥1% and the high level of expression of PD-L1 (PD-L1–H) as ≥5%, since very few cases occur with >50% overexpression [26,29,47]. These studies also reported a similar overall incidence of PD-L1 for patients in stage II/III CRC as in our study.

Although some studies suggest that PD-L1 expression is a negative prognostic factor, this is mainly due to the assessment of expression in tumor cells [33,58,59] and tumor staging. The contradictory results from the metastatic setting and from the early-stages [60] are probably due to temporal and spatial differences in the microenvironment and PD-L1 expression [61,62,63,64].

Patients with dMMR express significantly higher levels of PD-L1 in the early stages [26,47,48,65,66], which is consistent with the findings of our study (*p* = 0.043 for PD-L1–L and *p* < 0.001 for PD-L1–H). With respect to survival, patients in the CMS1 group, defined by dMMR, have the best prognosis in early-stage CRC [2,29,67,68] independent of the degree of PD-L1 expression. Further, the value of PD-L1 expression as an immunohistochemical biomarker of good prognosis when assessed in immune cells has been suggested by several studies and meta-analyses [33,47,63,69]. It is independent of MMR status [28,29,67,70,71]. Thus, patients with positive PD-L1 expression in the CMS2/CMS3 or CMS4 groups might also be expected to have a better prognosis.

This probably explains why our findings suggest that PD-L1 can separate those patients in the CMS2/CMS3 group with good and bad prognoses, since positive PD-L1 expression is significantly associated with better prognosis, as illustrated by the Kaplan–Meier curves for OS and DFS. This analysis was not carried out in the CMS4 group given the small number of statistical events upon which to draw relevant conclusions.

These results seem to be valid for other advanced GI tumors in general. Some recently published data suggest that PD-L1 expression has prognostic and predictive value and patients are being considered for anti-PD-1/PD-L1 therapy in CRC and other solid tumors [18,52,53,72,73,74].

As mentioned previously, CMS1 and CMS4 show very different intrinsic biological characteristics that translate into better and worse patient prognosis, respectively, in early-stage CRC [2,67,68]; this is not so clear for CMS2 and CMS3. As noted above, CMS2 displays epithelial differentiation and strong upregulation of WNT and MYC downstream targets, and CMS3 is characterized by multiple metabolism signatures. However, they sometimes share these characteristics with CMS4 or with CMS1 without distinction and this may be the reason for their unclear or intermediate prognosis [68]. For example, CMS2 shares with CMS4 a high frequency of somatic copy-number alterations and WNT/MYC pathways, and shares with CMS1 PD-1 activation and immune cell infiltration, whereas CMS3 shares with CMS4 higher KRAS mutation rates and sugar metabolic signatures, and shares with CMS1 a hypermutated profile and caspase pathways [3,19,75]. The results of our study could be a first step towards integrating the use of biomarkers like PD-L1 expression to differentiate the prognosis in CMS2 and CMS3. As such, it may significantly help with the clinical relevance of this classification.

Adjuvant chemotherapy in stage II and stage III CC patients remains controversial. For stage II despite several randomized trials [76,77], there is still a need for robust evidence concerning the addition of adjuvant chemotherapy for all patients [68]. For stage III, some studies have been able to establish a basis for treatment decisions [56,78,79]. Overall, some early-stage CRC patients benefit from adjuvant chemotherapy, although their long-term response rate is still suboptimal, particularly in the elderly population [68,80]. According to the ESMO and NCCN guidelines, the TNM is the main factor when deciding between observation or chemotherapy treatment. Nevertheless, other histopathological and clinical factors are sometimes taken into consideration, even though their prognostic value has not been fully validated [16,17].

Therefore, further characterization of patients with clinical implications is urgently needed in the context of early-stage settings. Our results, with PD-L1 expression used as a biomarker in combination with the CMS classification, could be a response to this need and possibly help with the decision to provide adjuvant therapy in the early setting.

Certain limitations of this study should be acknowledged when interpreting our results. Firstly, there were relatively few mortality events during the follow-up period, as expected during the design of the study. We used DFS because it is a good indicator in the Kaplan–Meier curves when mortality events are limited. Secondly, we assumed treatment to be homogeneous among all patients during the full course of their disease, since it was established and monitored by the same cross-functional committee of the same hospital. However, the lack of consistent data across patient records regarding the full details of the treatments received, treatment dosage, treatment duration, and/or any modifications, meant that the design of the study could not accommodate treatment stratification. Finally, the known limitations for a single center and retrospective study should also be acknowledged.

As mentioned previously, there is a clear need for better tools and characterization strategies for early-stage CRC patients. The early-stage setting has been less widely studied than the metastatic setting, probably due to its complexity and variability, even though the overall benefits to patients could be greater. With current emerging data and newly available targeted therapies, we call for a continuation of efforts towards devising validated prognostic biomarkers. Furthermore, a multi-center prospective study should follow our findings to confirm a hypothesized predictive value of PD-L1 expression.

## 5. Conclusions

In conclusion, our study demonstrates that PD-L1 expression is an independent prognostic factor in patients with stage II/III colon cancer in the CMS2/CMS3 group. The PD-L1 expression of stromal-related immune cells (tumor microenvironment) in colon cancer (CC) provides valuable information of prognostic value. The CMS classification itself is also of prognostic utility for early-stage CC patients. The assessment of CMS and PD-L1 expression through IHC, when performed in early-stage CC patients, may also have predictive value, with the potential to guide physicians concerning the addition of adjuvant treatment.

We expect this study to be a first step towards integrating the use of biomarkers like PD-L1 expression into a unified IHC panel, which may significantly help with the clinical relevance and implementation of the CMS classification.

## Figures and Tables

**Table 1:** Demographic and pathological characteristics.

Variable			N (%) *n* = 144
Age (years) *			72.2 (9.6)
		Range	48–93
Gender			
		Female	46 (31.9)
		Male	98 (68.1)
Localization			
		Right	79 (54.9)
		Left	65 (45.1)
Differentiation grade			
		<50%	26 (18.1)
		≥50%	118 (81.9)
Lymph node ratio			
		Mean * (SD)	6.7 (12.1)
		Median (Q1–Q3)	0.0 (0–9.3)
Histologic type			
		Colloid	18 (12.5)
		Adenocarcinoma	125 (86.8)
		Signet ring cell carcinoma	1 (0.7)
TNM Stage			
		II	80 (55.6)
		III	64 (44.4)
Lymphatic vascular invasion			
		Negative	108 (75.0)
		Positive	36 (25.0)
Blood vessel invasion			
		Negative	102 (70.8)
		Positive	42 (29.2)
Perineural invasion			
		Negative	112 (77.8)
		Positive	32 (22.2)

* Values are means. SD: Standard deviation. Q1–Q3: Quartiles 1–3.

**Table 2:** Prevalence of study variables.

Variable			N (%) *n* = 144
MMR status			
		pMMR	126 (87.5)
		dMMR	18 (12.5)
IHC BRAF (V600 mutation) *			
		Negative	125 (89.3)
		Positive	15 (10.7)
CMS classification			
		CMS1	18 (12.5)
		CMS2/CMS3	117 (81.3)
		CMS4	9 (6.3)
IHC PD-L1 expression			
		Negative	64 (44.4)
		≥1–<5%	51 (35.4)
		≥5%, >50%	29 (20.1)

* Not assessable in 4 tumor samples. p/dMMR: Mismatch repair proficient or deficient. CMS: Consensus Molecular Subtype. IHC: Immunohistochemical.

**Table 3:** Comparative analysis.

Variable		MMR pMMR *n* (%) dMMR *n* (%)	*p*	CMS1 *n* (%)	CMS2/CMS3 *n* (%)	CMS4 *n* (%)	*p*	PD-L1–L Neg (%) Pos (%)	*p*	PD-L1–H Neg (%) Pos (%)	*p*
Age	Mean (SD)	72.2 (9.5)		72.3 (10.6)	72.6 (9.2)	66.0 (11.2)		72.7 (10.4)		71.8 (9.6)	
		72.3 (10.6)	0.948 ^1^				0.134 ^1^	71.8 (9.0)	0.583 ^3^	73.9 (9.4)	0.296 ^3^
Gender	Men	86 (68.3)		12 (66.7)	79 (67.5)	7 (77.8)		41 (64.1)		77 (67.0)	
		12 (66.7)						57 (71.3)		21 (72.4)	
	Women	40 (31.7)		6 (33.3)	38 (32.5)	2 (22.2)		23 (35.9)		38 (33.0)	
		6 (33.3)	0.893 ^2^				0.884 ^4^	23 (28.7)	0.358 ^2^	8 (27.6)	0.573 ^2^
Localization	Right	63 (50.0)		16 (88.9)	57 (48.7)	6 (66.7)		29 (45.3)		61 (53.0)	
		16 (88.9)						50 (62.5)		18 (62.1)	
	Left	63 (50.0)		2 (11.1)	60 (51.3)	3 (33.3)		35 (54.7)		54 (47.0)	
		2 (11.1)	**0.002** ^2^				**0.003** ^4^	30 (37.5)	**0.039** ^2^	11 (37.9)	0.412 ^2^
TNM Stage	II	68 (54.0)		12 (66.7)	67 (57.3)	1 (11.1)		30 (46.9)		58 (50.4)	
		12 (66.7)						50 (53.1)		22 (75.9)	
	III	58 (46.0)		6 (33.3)	50 (42.7)	8 (88.9)		34 (62.5)		57 (49.6)	
		6 (33.3)	0.310 ^2^				**0.016** ^4^	30 (37.5)	0.061 ^2^	7 (24.1)	**0.014** ^2^
Lymphatic vascular invasion	No	92 (73.0)		16 (88.9)	87 (74.4)	5 (55.6)		47 (73.4)		83 (72.2)	
		16 (55.9)						61 (76.3)		25 (86.2)	
	Yes	34 (27.0)		2 (11.1)	30 (25.6)	4 (44.4)		17 (26.6)		32 (27.8)	
		2 (11.1)	0.243 ^4^				0.169 ^4^	19 (23.8)	0.699 ^2^	4 (13.8)	0.119 ^2^
Blood vessel invasion	No	88 (69.8)		14 (77.8)	90 (68.4)	8 (88.9)		40 (62.5)		79 (68.7)	
		14 (77.8)						62 (77.5)		23 (79.3)	
	Yes	38 (30.2)		4 (22.2)	37 (31.6)	1 (11.1)		24 (37.5)		36 (31.3)	
		4 (22.2)	0.488 ^2^				0.373 ^4^	18 (22.5)	**0.049** ^2^	6 (20.7)	0.261 ^2^
Perineural invasion	No	96 (76.2)		16 (88.9)	91 (77.8)	5 (55.6)		47 (73.4)		85 (73.9)	
		16 (88.9)						65 (81.3)		27 (93.1)	
	Yes	30 (23.8)		2 (11.1)	26 (22.2)	4 (44.4)		17 (26.6)		30 (26.1)	
		2 (11.1)	0.363 ^4^				0.146 ^4^	15 (18.8)	0.262 ^2^	2 (6.9)	**0.026 ^2^**
BRAF IHC *	Wild	118 (94.4)		7 (46.7)	109 (94.0)	9 (100)		56 (90.3)		105 (93.8)	
		7 (46.7)						69 (88.5)		20 (71.4)	
	Mutant	7 (5.6)		8 (53.3)	7 (6.0)	0 (0.0)		6 (9.7)		7 (6.2)	
		8 (53.3)	<**0.001** ^4^				**<0.001** ^4^	9 (11.5)	0.724 ^2^	8 (28.6)	**0.002** ^2^
MMR status	pMMR	-		0 (0.0)	117 (100)	9 (100)		60 (93.8)		107 (93.0)	
								66 (82.5)		19 (65.5)	
	dMMR	-		18 (100)	0 (0.0)	0 (0.0)		4 (6.3)		8 (7.0)	
			-				**<0.001** ^4^	14 (17.5)	**0.043** ^2^	10 (34.5)	**0.001** ^2^
PD-L1–L	Neg	60 (47.6)		4 (22.2)	58 (49.6)	2 (22.2)		-		-	
		4 (22.2)									
	Pos	66 (52.4)		14 (77.8)	59 (50.4)	7 (77.8)		-		-	
		14 (77.8)	**0.043** ^2^				**0.038 ^4^**		-		-
PD-L1–H	Neg	107 (84.9)		8 (44.4)	99 (84.6)	8 (88.9)		-		-	
		8 (44.4)									
	Pos	19 (15.1)		10 (55.6)	18 (15.4)	1 (11.1)		-		-	
		10 (55.6)	<**0.001** ^4^				**<0.001 ^4^**		-		-

^1^ ANOVA, ^2^ Chi-square test, ^3^ t-test, ^4^ Fisher’s exact test, two-tailed, ^5^ *t*-test. * Not assessable in 4 tumor samples. p/dMMR: Mismatch-repair proficient or deficient. CMS: Consensus Molecular Subtype. IHC: Immunohistochemical. Neg/Pos: Negative/Positive. SD: Standard deviation. Statistically significant *p* values are presented in **bold**.

**Table 4:** Univariate analysis of overall survival.

Variable (Reference)	Hazard Ratio	95% CI	*p* Value
Age (Mean)	1.09	1.04–1.14	**<0.001**
Gender (Male/Female)	0.96	0.46–2.00	0.913
Localization (Right/Left)	0.78	0.39–1.55	0.479
Stage (II/III)	1.51	0.76–2.99	0.242
Lymphatic vascular invasion	0.88	0.40–1.96	0.763
Blood vessel invasion	1.81	0.86–3.78	0.114
Perineural invasion	2.20	0.99–4.90	**0.050**
BRAF IHC * (wt/mutant)	0.25	0.05–1.14	0.056
MMR status (p/d)	0.48	0.15–1.54	0.211
CMS1–CMS2/CMS3	1.96	0.61–6.34	
CMSCMS1–CMS4	4.38	0.78–24.5	0.224
PD-L1–L	0.40	0.20–0.81	**0.010**
PD-L1–H	0.41	0.15–1.07	0.064

* Not assessable in four tumor samples. p/dMMR: Mismatch repair proficient or deficient. CMS: Consensus Molecular Subtype. IHC: Immunohistochemical. Statistically significant *p* values are presented in **bold**.

## Data Availability

Full data for this study are available from the Corresponding Authors upon request.

## Supplementary Materials

The following are available online at https://www.mdpi.com/article/10.3390/cancers13081943/s1, Figure S1: Scanned image of TMA (hematoxylin-eosin stain), Figure S2: IHC scan detail on high power field of PD-L1 staining in immune cells (core X20).
