# Supplementary material for: PD-L1 as a Prognostic Factor in Early-Stage Colon Carcinoma within the Immunohistochemical Molecular Subtype Classification
Source: Cancers (Basel). 2021 Apr 17;13(8):1943. doi: 10.3390/cancers13081943 (PMC8073668; doi:10.3390/cancers13081943)
Supplement: Supplementary file 1 [file cancers-13-01943-s001.zip › cancers-1181226-supplementary.pdf]

Article

Supplementary Materials: PD-L1 as a Prognostic Factor in Early-Stage Colon Carcinoma within the Immunohistochemical Molecular Subtype Classification

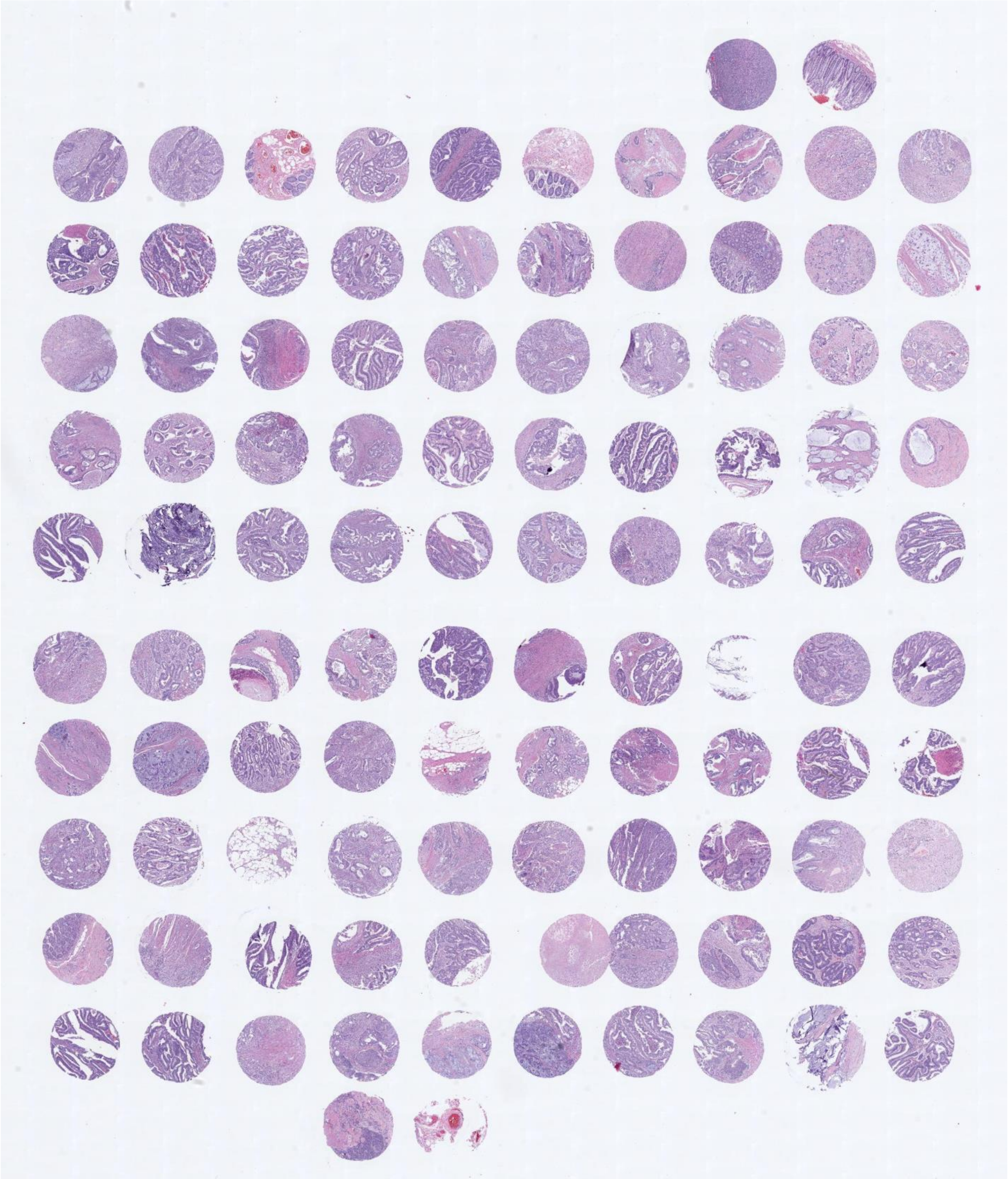

Figure S1. Scanned image of TMA (hematoxylin-eosin stain).

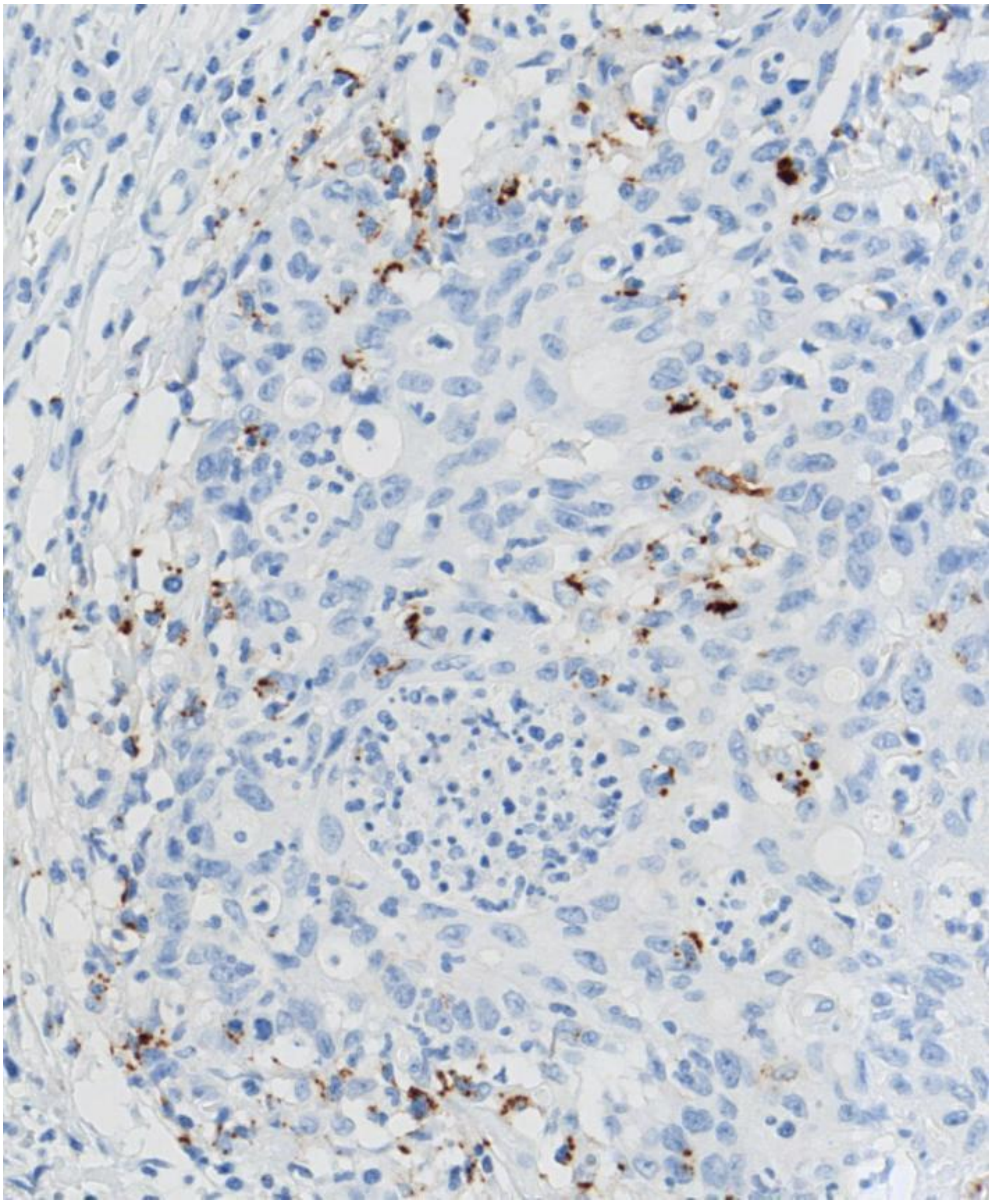

(a)

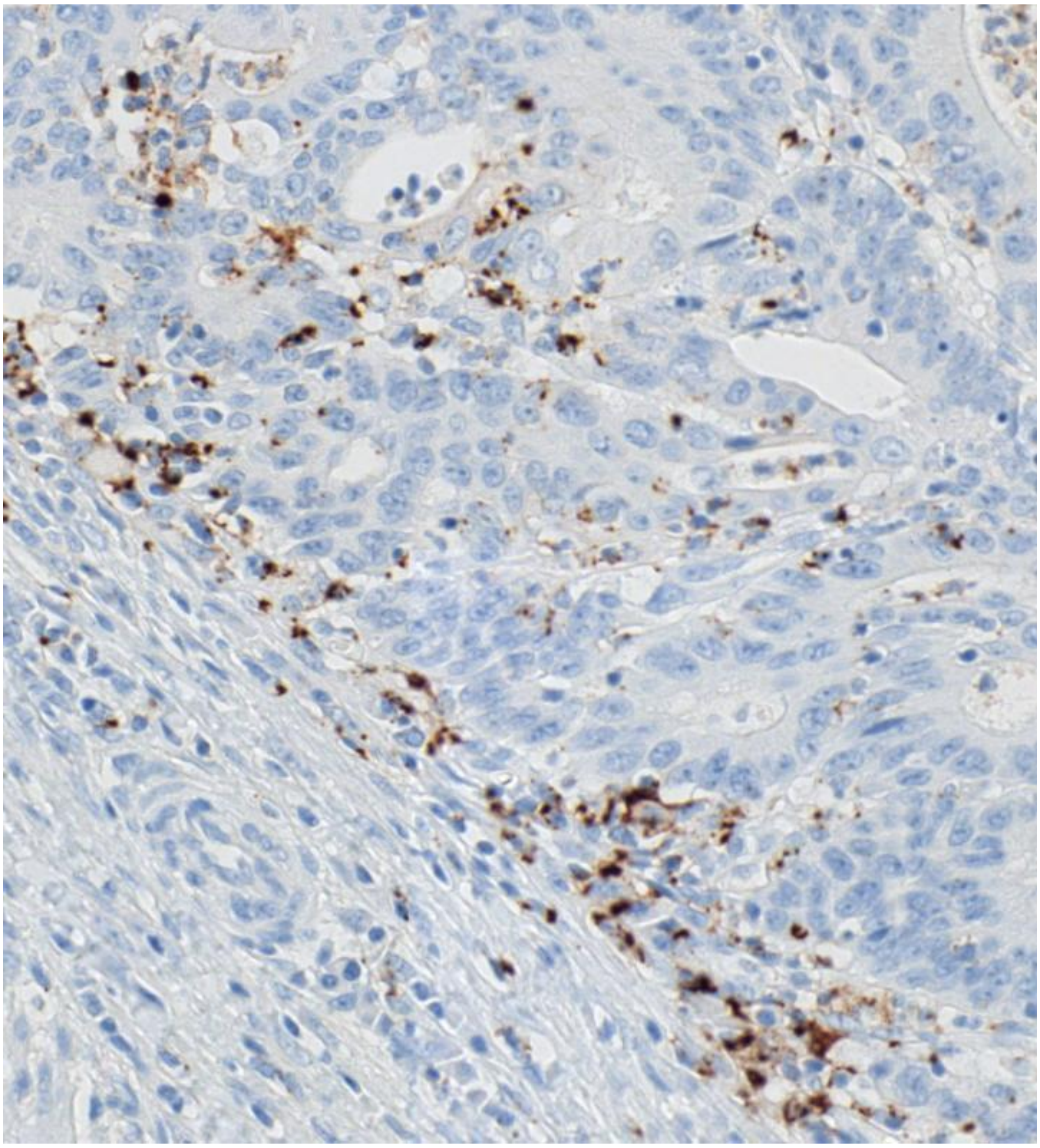

(b)

Figure S2. IHC scan detail on high power field of PD-L1 staining in immune cells (core X20).
